# Longitudinal genetic analyses of *Staphylococcus aureus* nasal carriage dynamics in a diverse population

**DOI:** 10.1186/1471-2334-13-221

**Published:** 2013-05-16

**Authors:** Gowrishankar Muthukrishnan, Ryan P Lamers, Austin Ellis, Vanathy Paramanandam, Alana B Persaud, Sergio Tafur, Christopher L Parkinson, Alexander M Cole

**Affiliations:** 1 Department of Molecular Biology and Microbiology, Burnett School of Biomedical Sciences, University of Central Florida College of Medicine, 4000 Central Florida Boulevard, Orlando, FL, 32816, USA; 2 Current affiliation: Department of Biochemistry and Biomedical Sciences, Michael G. DeGroote Institute for Infectious Disease Research, McMaster University, Hamilton, Ontario, Canada; 3 Stokes Advanced Research Computing Center, Institute for Simulation and Training, University of Central Florida, 3100 Technology Parkway, Orlando, FL, 32826, USA; 4 Department of Biology, University of Central Florida, 4000 Central Florida Boulevard, Orlando, FL, 32816, USA

**Keywords:** Staphylococcus aureus, Bacterial genetics, Multi locus sequence typing, Nasal colonization, *spa* typing, MRSA, *SCCmec* typing

## Abstract

**Background:**

*Staphylococcus aureus* (*SA*) nasal colonization plays a critical role in the pathogenesis of staphylococcal infections and *SA* eradication from the nares has proven to be effective in reducing endogenous infections. To understand *SA* nasal colonization and its relation with consequent disease, assessment of nasal carriage dynamics and genotypic diversity among a diverse population is a necessity.

**Results:**

We have performed extensive longitudinal monitoring of *SA* nasal carriage isolates in 109 healthy individuals over a period of up to three years. Longitudinal sampling revealed that 24% of the individuals were persistent *SA* nasal carriers while 32% were intermittent. To assess the genetic relatedness between different *SA* isolates within our cohort, multi locus sequence typing (MLST) was performed. MLST revealed that not only were strains colonizing intermittent and persistent nasal carriers genetically similar, belonging to the same clonal complexes, but strain changes within the same host were also observed over time for both types of carriers. More highly discriminating genetic analyses using the hypervariable regions of staphylococcal protein A and clumping factor B virulence genes revealed no preferential colonization of specific *SA* strains in persistent or intermittent carriers. Moreover, we observed that a subset of persistent and intermittent carriers retained clinically relevant community-acquired methicillin-resistant *SA* (CA-MRSA) strains in their nares over time.

**Conclusions:**

The findings of this study provides added perspective on the nasal carriage dynamics between strains colonizing persistent and intermittent carriers; an area currently in need of assessment given that persistent carriers are at greater risk of autoinfection than intermittent carriers.

## Background

*Staphylococcus aureus (SA)* is a leading cause of community-acquired and nosocomial bacterial infections in humans. *SA* infections can range from mild skin infections to severe, highly invasive and necrotizing diseases [1]. With the spread of community-acquired methicillin-resistant *SA* (CA-MRSA) and vancomycin-resistant *SA* (VRSA) strains around the world, it has become even more pertinent to conduct *SA* epidemiological studies to monitor its dissemination [2,3].

The most common niche of *SA* in humans is the anterior nares [4-6] and *SA* nasal colonization is thought to be a major source of bacterial transmission with *SA* colonizing approximately 25% of the human population asymptomatically [7-9]. *Staphylococcus aureus* nasal colonization has been attributed to an amenable host, and numerous epidemiological studies have been conducted to identify nasal carriers and non-carriers of *SA*[9-11]. However, to understand better the dynamics of *SA* nasal carriage over time, longitudinal studies are required. Nasal carriage patterns amongst healthy individuals can be broadly classified as persistent (always colonized by *SA* in their nares), intermittent or non-carriers [9,12,13]. This distinction is important as persistent carriers are at a higher risk of developing active auto-infections than intermittent and non-carriers [9,11,14,15].

To understand better the genetic diversity of *SA* strains that colonize nasal carriers, the population structure of *SA* strains obtained from healthy individuals must be defined and detailed. Multi locus sequence typing (MLST) is one of the most common means by which population structure of *SA* strains have been analyzed [16-18]. More recently, genotyping of hypervariable virulence genes (staphylococcal protein A (*spa*) [19,20] and clumping factor B (*clfB*) [21]) have also been employed to enhance strain resolution and thus offer better characterization of genetic relatedness between *SA* strains. Moreover, with the increasing prevalence of CA-MRSA, it is critical to understand the origin and the dissemination of major MRSA clones within the healthy population [22-25]. SCC*mec* typing, the most common means by which to identify MRSA, has become a vital tool for the characterization of CA-MRSA clones in epidemiological studies [26,27].

Several studies including ours [28,29] have shown that *SA* nasal colonization is multifactorial, involving not only bacterial determinants but also host factors that predispose individuals to *SA* carriage [7,30-35]. However, the exact mechanisms leading to persistent versus intermittent or non-carriage remain unclear. It is also unknown whether persistent and intermittent hosts preferentially carry a specific genotype of *SA* strains. Therefore, understanding the patterns of nasal carriage and the preferential colonization by certain genotypes of *SA* strains in persistent and intermittent carriers will greatly augment our understanding of *SA* nasal carriage.

Recently, we revealed genetic associations between nasal carriage strains and clinical isolates in a cross-sectional survey of healthy individuals [36]. In the current study, we extended these analyses and longitudinally assessed the population structure of *SA* nasal carriage strains in a diverse population for a period of up to three years to gain a better understanding of nasal carriage dynamics, in addition to assessing whether preferential colonization by certain genotypic *SA* strains occurs within persistent versus intermittent carriers. Interestingly, MLST analyses revealed that both intermittent and persistent carriers harbor genotypically similar strains that cluster into the same clonal complexes. Furthermore, these strains exhibited similarity to *SA* isolates of clinical significance. Genotyping studies using housekeeping (MLST) and hypervariable virulence genes (*spa* and *clfB*) revealed that both persistent and intermittent carriers change strains over time with no difference in the frequency of strain change between the two carrier groups. The current study contrasts previous findings that have indicated that persistent carriers carry the same *SA* strain over long periods of time while intermittent carriers carry different strains during *SA* nasal carriage [9,37]. Overall, this study indicates that colonizing strains of *SA* are not specific to a particular host or carriage type (i.e., persistent versus intermittent carriers) and both carriage type change strains over time, suggesting that other non-*SA* factors could be contributing to specific carriage states.

## Methods

### Ethics statement for collection of bacterial strains from donors

The current study was approved by the University of Central Florida’s Institutional Review Board (UCF IRB). All donors provided informed written consent to participate in the current study. Nasal swab sample collection for the current study was undertaken in the University of Central Florida (UCF) campus. UCF is a diverse community of nearly 60,000 students and approximately 8000 faculty and staff members of various ages, ethnic and racial backgrounds. All procedures and investigators involved in the sample collection process were Institutional Review Board (IRB)-approved with Collaborative Institutional Training Initiative (CITI) certification.

### Study population, design and bacterial strains

A total of 329 healthy individuals at UCF were screened for the presence of *SA* in their anterior nares. Specifically, the donor population (58.35% - Female, 40.72% - Male and 0.93% - Unreported) consisted of participants from various racial and ethnic backgrounds (White -56.84%, Asian – 13.07%, Black – 17.63%, Pacific Islanders – 1.22%, Hispanic/Latino – 13.07%). Of the 329 individuals screened, 96 (29.2%) tested positive for *SA* nasal colonization at least once while the remaining 233 (70.8%) donors were classified as non-carriers because *SA* was never isolated from their nares. Of the 329 total individuals enrolled in our study, 109 participants – comprised of 61 carriers and 48 non-carriers – were monitored longitudinally (i.e., multiple nasal swab samples were collected from these individuals). Among the 96 *SA* positive carriers, 61 were monitored longitudinally while the remaining 35 carriers were screened for nasal colonization only once. In total, a median of four (range 2–18) nasal samples were obtained from each of 109 healthy individuals (including individuals that tested negative for *SA*) for a varying period of up to three years, with duration and frequency of collections dependent on donor availability. Following screening, donors were classified into persistent (if all nasal cultures tested *SA* positive for the duration of the study), intermittent (if at least one nasal culture tested negative for *SA* over the course of the study), and non-carriers (no cultures tested positive for *SA*) of *SA*.

Following nasal sample collection, *SA* strains were isolated as previously described [36]. Briefly, the anterior nares of the donors were swabbed with sterile, unflocked polyester-tipped swabs (Fisher Scientific, Pittsburgh, Pennsylvania, USA) and nasal samples were grown overnight on nutrient rich Tryptic Soy Agar (TSA) supplemented with 5% sheep’s blood (Becton, Dickinson and Company, Franklin Lakes, New Jersey, USA). Bacterial colonies were identified as *SA* using Staphyloslide™ Latex Test reagent (Becton, Dickinson and Company, Franklin Lakes, New Jersey, USA) and sub-cultured in Trypticase Soy Broth (TSB; Becton, Dickinson and Company, Franklin Lakes, New Jersey, USA) overnight at 37°C and 250 rpm. Overnight cultures were subsequently used for isolation of genomic DNA.

### Multi locus sequence typing

Genomic DNA from *SA* isolates was extracted using GenElute™ Bacterial Genomic DNA kit (Sigma-Aldrich Co., St. Louis, Missouri, USA), according to the manufacturer’s instructions. Following extraction, multi locus sequence typing (MLST) of seven housekeeping genes *(arcC, aroE, glpF, gmk, pta, tpi, and yqiL)* was performed using primers and PCR conditions as previously described [17,36]. Sequence types (STs) for each *SA* strain were obtained based on the alleles identified at each of the seven loci using the *SA* MLST database (http:// http://www.mlst.net). New alleles and STs were submitted to the MLST database curator and subsequently added to the database.

### Phylogenetic analyses of MLST data

Phylogenetic analysis of the concatenated MLST data of all isolates was performed as previously described [36] using the Metropolis-Hastings coupled Markov chain Monte Carlo method (MCMC) implemented in MrBayes v3.1.2 [38-40]. Triplicate MCMC analyses were performed in parallel [40] using the STOKES IBM High Performance Computing Cluster at UCF. Bayesian MCMC analyses were carried out using both partitioned and unpartitioned concatenated MLST data. Best-fit evolutionary models for each individual gene fragment (in the partitioned dataset) as well as unpartitioned dataset were selected based on Akaike Information Criterion implemented in jModelTest v0.1.1 [38,41]. For the concatenated unpartitioned MLST dataset, a generalized time-reversible (GTR) evolutionary model with inverse-gamma distribution was selected as the best-fit model. For loci *glpF*, *pta* and *yqiL* in the partitioned dataset, the Hasegawa, Kishino and Yano (HKY) substitution model was chosen while a HKY model with a gamma distribution was chosen for the *arcC* gene [42]. Additionally, the HKY model including invariable sites (HKY + I) was selected for locus *gmk*. For the *tpi* locus, a GTR substitution was the chosen model while a GTR + I model was identified as the best-fit substitution model for the *aroE* locus. Within each replicate MCMC analysis two independent Bayesian runs were performed with random starting trees and default settings. Each run consisted of 5 million generations with every 100 steps being sampled. For each analysis, a steady stationary state of the run was verified using Tracer v1.5 and a burn-in of 25% of the generations was performed. A final run consisting of 20 million generations was also performed to verify the likelihood scores from the shorter runs were consistent with the longer runs.

### eBURST analyses of MLST data

The different Sequence types (STs) that were identified for each *SA* strain were classified into different groups using the eBURST v3 analysis software [43,44]. Each ST was assigned to a cluster group requiring six of the seven loci between members of the group to be identical [44]. eBURST analysis was also used to assess relatedness of nasal carriage strains to nosocomial epidemic strains.

### ***spa*** typing and eBURP

*SA* isolates were *spa* genotyped using primers and PCR conditions described previously [19,20] and sanger sequenced [45] at Eton Bioscience Inc. DNA sequencing facility (Durham, North Carolina, USA). *spa* types were determined using the Ridom StaphType (Ridom GmbH) software (http://www.spaserver.ridom.de/). All *spa* types including those newly identified were synchronized with the global *spa* type database via the StaphType server. To partition the intermittent and persistent carriers, eBURP-clustering analysis using the Ridom StaphType software was performed using default settings. *SA* isolates having less than 5 repeat units were excluded from the clustering analysis, as it is difficult to infer evolutionary history of a *SA* strain from spa type with less than five repeat units [20].

### ***clfB*** typing and sequence

For all *SA* isolates, the hypervariable region of the *clfB* gene was amplified and sequenced using the protocols and primers described previously [46,36]. Subsequently, sequence analyses of the hypervariable repeat region was performed using the in-house sequence analysis software described previously [36]. Briefly, the nucleotide sequence of the R region of *clfB* gene was converted into a numeric profile based on the unique repeat units (Additional file 1: Table S3). Subsequently, each unique repeat unit was assigned a specific color-coded box and the numeric output profile of clfB R region was converted into a color-coded representation (Additional file 2: Figure S3) [36].

### ***SCCmec*** typing

*SA* isolates were also screened for the presence of the *SCCmec* gene cassette that confers resistance to the antibiotic methicillin. Phenotypic screens for MRSA strains were performed by streaking single *SA* colonies on selective chromogenic MRSASelect™ agar plates (Bio-Rad, Hercules, CA, USA) and identified following the manufacturer’s instructions. Following the phenotypic screening, a multiplex PCR reaction amplifying eight different loci of the *SCCmec* gene cassette was performed on the MRSA strains to determine the type assignment of the *mec* gene. The primers, protocols, and analyses used for multiplex PCR were performed as previously described [26,27].

### Statistical analysis

Student’s t-tests for the differences in the length of *clfB* R region and X domain repeat region of *spa* gene were conducted using GraphPad Prism 4 software (GraphPad Software, La Jolla, CA, USA). A 2 × 2 contingency table was constructed and a G-test was performed to analyze the distribution of persistent and intermittent carriers among males and females within the cohort. Similarly, a 2 × 2 contingency table was constructed to evaluate the trend of *SA* strain change between persistent and intermittent carriers and a G-test was performed to assess the differences in strain change in persistent and intermittent carriers. G-tests were performed using JMP Pro software (SAS Institute Inc., Cary, NC, USA) [47].

## Results

### Longitudinal assessment of *SA* nasal colonization in a healthy population identified persistent and intermittent carriers

**Figure 1:**
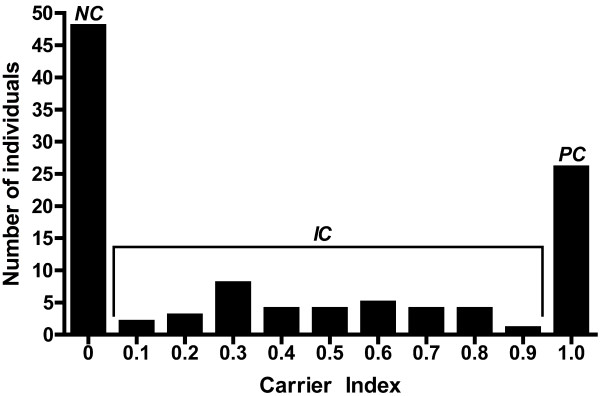
**Distribution of *SA *nasal carrier indices among 109 healthy individuals monitored longitudinally.** Carrier index is defined as the number of *SA* positive nasal swabs over number of total swabs for each individual person. A total of 61 *SA* nasal carriers and 48 non-carriers were monitored longitudinally and their respective carrier indices are represented here. *NC*, indicates *SA* non-carrier state; *SA* intermittent carriage state; and *PC*, *SA* persistent carriage state.

To assess nasal colonization state over time, extensive longitudinal monitoring was performed in which multiple nasal samples were obtained from 109 healthy individuals for a period of up to three years. Following longitudinal sampling, donors were classified into persistent, intermittent, and non-carriers of *SA* based on their carrier indices (defined as the number of *SA* positive nasal swabs over the total number of swabs for each individual; Figure 1) such that all non-carriers and persistent carriers have carrier indices of exactly 0 and 1 respectively, while intermittent carriers have scores *between* 0 and 1. In total, sixty-one (56%) individuals were *SA* nasal carriers (23.8% persistent and 32.1% intermittent) and 48 (44.0%) were non-carriers. Within the study population, 23.8% of all female donors were persistent carriers while 30.1% were intermittent and 23.9% of all male participants were persistent carriers while 34.7% were intermittent (Likelihood ratio χ^2^ = 0.070, N = 61, degrees of freedom (df =1), *p* = 0.7911, Table 1). Our comprehensive longitudinal monitoring for *SA* nasal colonization revealed true persistent and intermittent carriers and subsequently, genotyping studies were conducted on the isolated *SA* strains to assess genetic relatedness among them.

### *SA* strains isolated from persistent and intermittent carriers belong to the same genetic clusters as nosocomial strains

We have recently revealed genetic associations between nasal carriage strains and clinical isolates [36]; however, this initial study was a static cross-sectional survey that did not account for the nasal carrier class of donors (i.e. persistent vs. intermittent). In the current study, we have extended these analyses to a larger cohort of donors, including persistent and intermittent carrier strains that were monitored longitudinally for a period of up to three years. To determine the genetic relatedness among *SA* strains, MLST analyses were performed on 297 *SA* nasal carriage strains obtained from 96 individuals. A total of 42 different sequence types (STs) were observed with 10 being newly identified (refer to Additional file 1: Table S1 for genotyping details of all *SA* strains used in this study). Three novel alleles were also identified in this study at loci *glpF*, *gmk* and *pta.* Sequence types 5 (21.3% of all carriers), 30 (18% of all carriers) and 8 (16.4% of all carriers) were the most prevalent STs observed within the cohort (Table 2). *Staphylococcus aureus* strains belonging to ST15 were only isolated from persistent carriers. However, only one of these persistent carriers was monitored for more than one year and as such, elaborate longitudinal monitoring of a larger cohort of donors containing ST15 *SA* strains is required to determine if there is any preferential colonization of persistent carriers by ST15 *SA* strains.

**Table 1:** Distribution of persistent and intermittent carriers among males and females

**Sex**	**Carriers (% carriage distribution across sex)**^**#**^			**Total**^**a**^
	**Persistent**	**Intermittent**	**Non-carriers**	
Male	11 (23.9)	16 (34.7)	19 (41.4)	**46**
Female	15 (23.8)	19 (30.1)	29 (46.1)	**63**
**Total**^**b**^	**26**	**35**	**48**	**109**

^#^Only nasal swabs from carriers sampled 2 or more times were only included.

^a^Total number of nasal carriers distributed across each sex.

^b^Total number persistent, intermittent and non-carriers monitored during the study.

**Figure 2:**
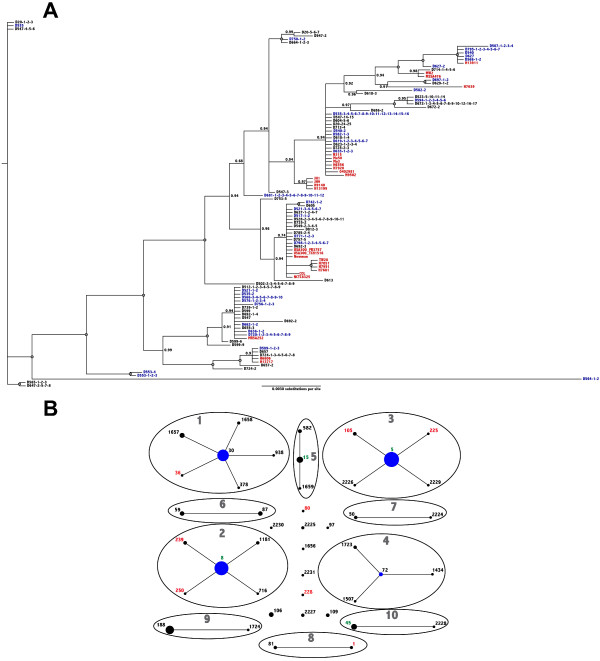
***SA *strains from persistent and intermittent nasal carriers are genetically related to nosocomial epidemic strains.** (**A**) Bayesian MCMC analysis of persistent carrier strains (colored in **blue**), intermittent carrier strains (colored in **black**) and nosocomial epidemic strains (colored in **red**). Numbers at each node indicate posterior probability support and grey-filled circles represent 100% posterior probability. (**B**) eBURST analysis of the MLST data clusters STs from intermittent and persistent carriers into same clonal complexes and into groups that are represented by numbers in **grey**. STs colored in **black** are nasal carrier strains, STs colored in **red** are epidemic strains and those in **green** contain both carrier and epidemic strains. Circle sizes in each cluster are proportional to the number of isolates and **blue** circles are founders of that particular cluster.

Bayesian MCMC analysis of the concatenated MLST data revealed that *SA* strains isolated from persistent and intermittent carriers are closely related. Persistent and intermittent carriers as well as strains isolated from clinical studies all group within the same clades (Figure 2A). Since the cohort included healthy individuals that were singly sampled (cross-sectional), phylogenetic analyses incorporating *SA* MLST data from these individuals were also performed (Additional file 3: Figure S1, Supplemental Information) and the analyses reveal that all *SA* carrier strains within the cohort are highly similar to strains of clinical origin.

In addition to identifying phylogenetic relationships, eBURST clustering of the MLST data confirmed that persistent and intermittent carrier strains belong to the same clonal complexes as that of epidemic strains. As observed in Figure 2B, eBURST delineated nasal carriage and clinical strains into 10 groups and 11 singletons. Of these, five groups contained both clinical and nasal carriage strains and groups identified by eBURST also contained STs from both persistent and intermittent carrier strains (data not shown). Collectively, the phylogenetic analyses revealed genetic relatedness between persistent and intermittent carrier strains, in addition to genetic similarities with strains isolated from clinical settings.

**Table 2:** Predominant STs in persistent and intermittent carriers

**Sequence type (ST) of *S. aureus *strains**^**#**^	**Number of donors carrying each ST (% of donors carrying each ST)**^**a**^		**Total**^**b**^
	**Persistent**	**Intermittent**	
ST5	5 (19.2)	8 (22.8)	**13 (21.3)**
ST30	5 (19.2)	6 (17)	**11 (18)**
ST8	4 (15.4)	6 (17)	**10 (16.4)**
ST45	1 (3.8)	2 (5.7)	**3 (4.9)**
ST15	4 (15.4)	0	**4 (6.5)**
ST59	1 (3.8)	2 (5.7)	**3 (4.9)**
ST188	1 (3.8)	3 (8.5)	**4 (6.5)**

^#^Only nasal swabs from carriers sampled 2 or more times were included and only the most prominent STs prevalent in North America are presented here.

^a^Percentage calculated using the total number of persistent and intermittent carriers in the cohort.

^b^Total number persistent and intermittent carriers that carried one or more strains in their noses.

### *SA* strains from the nares of both persistent and intermittent carriers change over time

**Figure 3:**
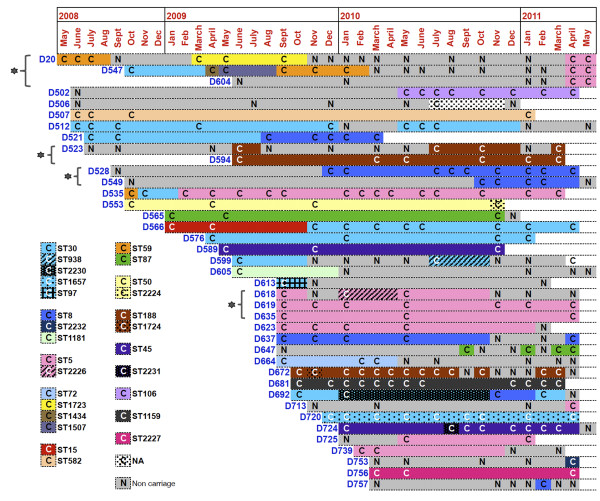
**Longitudinal monitoring reveals that *SA *strains from both persistent and intermittent nasal carriers change over time.** A representative set of persistent and intermittent carriers that have been monitored for at least one year is depicted here. (C) indicates *SA* nasal carriage at the time of swabbing and (N) indicates *SA* non-carrier state. Colors represented in the figure correspond to different Sequence Types (STs) identified by MLST. STs are segregated into different cluster groups by eBURST analysis. Carriers within the same household are grouped next to each other (indicated by * and flower bracket). (NA) corresponds to ST not available.

To date, few studies longitudinally assessed whether nasal carriage strains of *SA* in the nares of persistent and intermittent carriers change over time. Longitudinal monitoring of 61 carriers (both persistent and intermittent) revealed variations in the STs of *SA* nasal carriage strains over time. A representative set of persistent and intermittent carriers that were monitored between one and three years is depicted in Figure 3, revealing the patterns of strain change. In addition, 48 healthy individuals were monitored over time and identified as true non-carriers (see Additional file 4: Figure S2, Supplemental Information). Notably, it was observed that individuals who share households (such as spouses, siblings, roommates, etc.) tended to carry genetically similar strains (Additional file 1: Table S2). For example, family members and individuals living in the same households (D528-D549, D523-D594, D618-D619 and D20-D547-D604 (Figure 3)) carried genetically similar strains at one or more sampling times. Interestingly, we observed that intermittent carriers D523 and D618 harbored genetically similar strains as that of their living partners D594 and D619, respectively. Additionally, it was observed that persistent carriers D619 and D635, who are identical twins, carried genetically similar strains during the entire study period. Though additional correlative studies are required, interesting trends of *SA* transmission over time among individuals living in the same household were observed within the cohort.

Previous reports have indicated that a single strain of *SA* colonizes the nose for long periods of time in persistent carriers while strains colonizing intermittent carriers tend to exhibit more extensive genotypic diversity [9,37]. In contrast, within our cohort, we observed that over time, 27% of persistent carriers and 23% of intermittent carriers changed the ST of their *SA* strain (Likelihood ratio χ^2^ = 0.132, N = 61, df =1, *p* = 0.7160, Table 3). Additionally, phylogenetic analyses of the MLST data revealed that *SA* strains from these carriers clustered into the same genetic clades exhibiting a high degree of relatedness. Taken together, these results indicate similar genotypic diversities of colonizing strains in persistent and intermittent carriers.

### Genotyping of hypervariable virulence genes revealed no preferential colonization of either persistent or intermittent carriers by specific *SA* strain genotypes

As MLST is based on housekeeping genes that evolve slowly [18], we also genotyped hypervariable virulence genes (*spa* and *clfB*) in order to obtain higher levels of strain resolution and further characterize the relatedness among strains obtained from persistent and intermittent carriers. Genotyping of the virulence gene *spa* was performed on 242 *SA* strains isolated from persistent and intermittent carriers. A total of 41 unique *spa* types were obtained, nine of which were newly identified in this study. Interestingly, high sub-ST strain resolution was obtained at the *spa* locus (discriminatory index of 0.957), and 11 (26.83%) of the 41 *spa* types identified contained persistent and intermittent carrier strains exhibiting identical X domain repeats.

**Figure 4:**
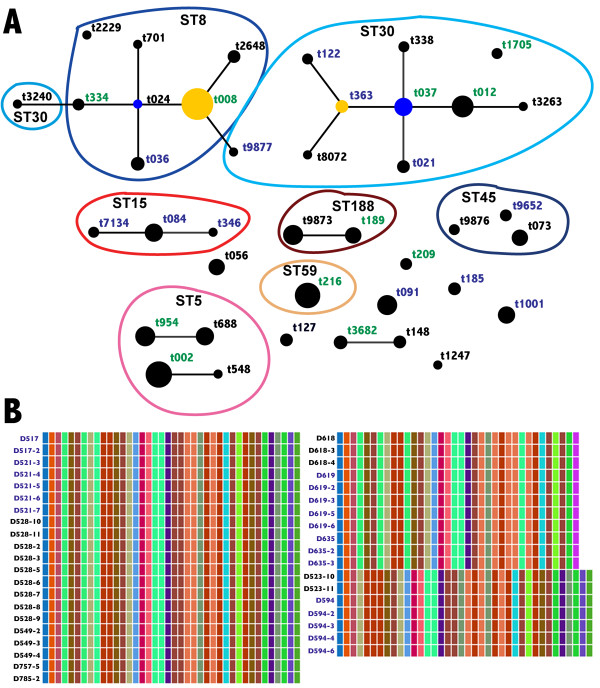
**Genotyping of hypervariable virulence genes revealed no preferential colonization of specific genotypes of *SA *strains in persistent and intermittent carriers.** (**A**) eBURP clustering analysis based on *spa* types revealed that both persistent and intermittent carrier strains belonged to same clonal complexes. *spa* types colored in **blue** contain only persistent carriers while those in **black** contain only intermittent carriers. *spa* types colored in **green** contain both intermittent and persistent carriers. Circle sizes in each cluster are proportional to the number of isolates and inferred founders (**blue** circles) and sub-founders (**yellow** circles) of each cluster are also represented here. *spa* types with less than 5 repeats were excluded from the eBURP analysis. (**B**) A representative set of *SA* persistent (colored in **blue**) and intermittent (colored in **black**) carrier strains having indistinguishable clfB R domain repeat region sequences. Like-colored boxes indicate 100% sequence similarity between *SA* strains.

eBURP-clustering analysis performed on the *SA* strains grouped them into seven clonal complexes (*spa*-CC) and 13 singletons (refer to Additional file 1: Table S1 for *spa* typing details of all *SA* strains used in this study). Interestingly, eBURP revealed that *spa* types from both persistent and intermittent carriers clustered into the same clonal complexes, confirming the high degree of genetic relatedness observed in MLST phylogenetic analyses (Figure 4A).

In addition to *spa* typing, we also performed genotyping of the hypervariable R region of *clfB.* This region determines the length of the extracellular ligand binding domain of ClfB protein, which is thought to influence bacterial adherence to host epithelia [48]. A previously developed in-house software was used to analyze this *clfB* R region [36]. Nucleotide analysis of the c*lfB* R region was performed on 244 *SA* strains isolated longitudinally, and a total of 109 unique repeat units were observed (Additional file 1**:** Table S3). Though variability was observed in the *clfB* gene fragments, 34.15% of all persistent carrier strains analyzed in our study contained identical sequence repeats to strains isolated from intermittent carriers, revealing relatedness between the *SA* strains. Figure 4B depicts the sequence similarity of the *clfB* repeat regions in a representative sampling of nasal carriage strains isolated from persistent and intermittent carriers. Refer to Additional file 2: Figure S3 for *clfB* typing details of all *SA* strains analyzed in this study.

**Table 3:** **Persistent and Intermittent carriers carrying more than one unique *SA *lineage in their noses during the study period**

**Number of different *S. aureus *strains**^**#**^	**Carriers (% of total in the carrier group)**		**Total**^**a**^
	**Persistent**	**Intermittent**	
1	19 (73)	27 (77)	**46 (75.4)**
2	6 (23)	4 (11.5)	**10 (16.4)**
3	1 (4)	3 (8.5)	**4 (6.6)**
4	0	0	**0**
5	0	1 (3)	**1 (1.6)**
**Total**^**b**^	**26**	**35**	**61**

^#^Nasal swabs from carriers monitored 2 or more times were only included.

^a^Percentage calculated using the total number of persistent and intermittent carriers in the cohort.

^b^Total number persistent and intermittent carriers that carried one or more strains in their noses.

**Figure 5:**
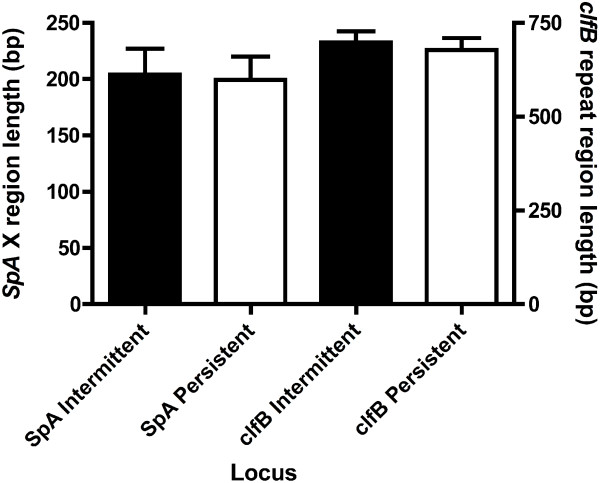
***spa *and *clfB *repeat domain lengths are indistinguishable between persistent and intermittent carriers.** Plots comparing X domain repeat region of *spa* and R region lengths of *clfB* between persistent and intermittent carrier *SA* strains.

Recently, human *in vivo* nasal colonization studies revealed ClfB exhibits a crucial function in bacterial adherence to the nares [32]. Therefore, we assessed whether differences in the length of *clfB* R region would correlate to intermittent or persistent carriage. Persistent carrier strains contained nearly identical R region lengths compared to intermittent carrier strains (*p* = 0.6646, Figure 5), suggesting that strains from these groups exhibit a high degree of relatedness. A similar analysis of the X domain repeat region of *spa* also revealed no significant difference in length between these two groups (*p* = 0.7797, Figure 5).

Collectively, longitudinal monitoring of *SA* nasal carriage strains followed by MLST and genotyping of hypervariable virulence genes (*spa* and *clfB*) revealed a high degree of genetic relatedness between *SA* strains colonizing persistent and intermittent carriers. These results indicate no preferential colonization of either persistent or intermittent carriers by certain genotypes of *SA*.

### Persistent and intermittent carriers harbor epidemic MRSA strains in their nares longitudinally over time

All 297 *SA* strains analyzed in this study were subjected to phenotypic screening to identify MRSA strains, and 11.78% of all *SA* carriers (sampled once or multiple times) carried MRSA strains in their nares. A subset of persistent and intermittent carriers harbored strains that were similar to CA-MRSA strains in their nares longitudinally over time (Table 4). Both occurrences of losing and acquiring MRSA strains were observed in these carriers throughout the colonization study period. Persistent carrier D798 carried an ST8-*SCCmec* type IV strain, which is genetically similar to the widely disseminated epidemic CA-MRSA strain USA300. Additionally, the persistent carrier D535 acquired and carried ST5-*SCCmec* type II MRSA strain for over two years. This strain is genotypically similar to another widespread nosocomial epidemic MRSA strain N315. These results indicate that some persistent and intermittent carriers carry epidemic MRSA strains in their nares over variable periods of time.

**Table 4:** **Classification of MRSA strains from persistent and intermittent carriers using *SCCmec *typing**

**Donor**	**MLST Sequence type (ST)**	***SCCmec *type**
**D535-2**^**a**^	ST30	I
**D535-3-4-5-6-7-8-10-11-12-13-14-15-16**	ST5	IV
*D547*^b^	ST30	I
*D565-1-3*	ST87	III
*D618*	ST30	IV
*D795-2*	ST15	II
**D798-1-2-3-4-5-6**	ST8	IV

^a^**Bold** indicates persistent MRSA carrier strains.

^b^*Italics* indicates intermittent MRSA carrier strains.

## Discussion

There is considerable evidence indicating that *SA* carriage is an important risk factor for endogenous infection, and recent studies have substantiated that *SA* nasal carriage is multi-factorial, involving both host and bacterial factors [7,9,28,49-51]. However, little is known about the extent to which the colonizing strains’ factors contribute to persistent versus intermittent carriage of *SA* in the human nose. Therefore, as one of our goals, we set out to investigate whether there is preferential colonization by particular genotypes of *SA* strains among persistent and intermittent carriers. We observed no preferential colonization by particular genotypes of *SA* strains during colonization of either persistent or intermittent carriers. These findings reveal the close genetic relatedness of *SA* strains carried by the carriers in our cohort and raise additional questions about other factors that are responsible for determining persistent versus intermittent carriage states. Previous studies suggest that host factors are crucial determinants of *SA* carriage [50,52] and the fact that this study could not find any genetic differences between strains colonizing persistent and intermittent carriers, collectively may imply as yet unknown factors (including host, microbiome and environment [53]) could primarily be responsible for determining carriage state.

The definition of persistent carriage varies between studies, and one study defined persistent carriage based on a semi-quantitative approach, called the “culture rule” where nasal swabs were collected one week apart to determine persistent or intermittent carriage [54]. However, it is arguable that a more comprehensive longitudinal sampling over longer periods of time is required to identify true persistent carriers. In the current study, extensive longitudinal monitoring of healthy individuals was performed for a period of up to three years to differentiate true persistent carriers from intermittent carriers and non-carriers. This distinction is crucial because bacterial loads between persistent and intermittent carriers vary widely (about 1000 fold more CFUs in persistent carriers [54]), which puts persistent carriers at a higher risk of acquiring *SA* infections [11,15]. Interestingly, we observed that some persistent carriers carry highly virulent epidemic CA-MRSA strains like USA300 in their nares longitudinally over time, potentially putting them at greater risks of acquiring MRSA infections. CA-MRSA clone USA300 is a widely disseminated virulent strain that is responsible for majority of community associated soft tissue and skin infections [55,56]. Though *SA* nasal carriage itself is seemingly benign to the host’s nose, carriers in general are known to require the use of antibiotics more than non-carriers (Rotterdam ERGO cohort [57]). More frequent antibiotic usage could lead to the emergence of multidrug resistant *SA* strains, in addition to affecting the equilibrium of the host’s commensal flora.

Previous studies have suggested that a single *SA* strain often colonizes the nose for long periods of time in persistent carriers while strains colonizing intermittent carriers tend to exhibit more genotypic diversity as periods of decolonization and recolonization occur [9,37]. In contrast, our longitudinal sampling and genotyping studies (using MLST, *spa* and *clfB*) revealed that *SA* strains carried by both persistent and intermittent carriers clustered into the same clades exhibiting high degree of genetic relatedness and *SA* strains carried in their nares change over varying periods of time. It is likely, however, that these changes are due to the acquisition of distinct strains—that are genetically similar to the one being replaced—as opposed to the same strain undergoing mutational events. While high sub-ST strain resolution and genotypic analyses of relatedness were obtained in this study, large scale whole genome sequencing of *SA* strains isolated from intermittent and persistent carriers may be the most accurate technique in discerning the genetic relatedness in these *SA* strains. Next generation sequencing technologies could surely assist with such large-scale genome studies [58].

Several hypervariable virulence genes like *spa* and *clfB* have been postulated to be involved in *SA* nasal carriage [7,29,30,32-35,59]. However, it is unclear whether polymorphisms in these genes and differences in their repeat lengths would affect the ability of *SA* to bind nasal epithelia and hence, contribute to persistent or intermittent carriage. Our longitudinal analyses revealed that strains isolated from persistent and intermittent carriers showed a high degree of genetic relatedness with respect to polymorphic changes in *spa* and *clfB* genes. These findings echo the findings of a previous study, which demonstrated that polymorphisms in repeat regions of virulent genes *spa* and *coa* (coagulase) do not contribute to persistent carriage [60]. In fact, no studies to date have been able to detect any bacterial factors involved in distinguishing persistent versus intermittent carriage states, suggesting a greater role for other factors in carriage type.

It has been previously speculated that the carriage state can be imposed on members of the same household [61,62]. The current study, though limited, also observed patterns of *SA* transmission among individuals living in the same household in which persistent and intermittent carriers cohabitating in the household harbored genetically similar *SA* strains. In a similar fashion, studies among the institutionalized elderly population observed that both persistent and intermittent carriage strains are shared among household members and the transmitted *SA* strains exhibited genotypic similarities [61]. However, additional correlative studies using a larger cohort of individuals living in the same household are necessary.

Bacterial interference has been hypothesized to be involved in determining *SA* non-carriage state rather than carriage state. Commensal flora of the body are known to protect the host against acquisition of new *SA* strains [63]. The phenomenon of bacterial interference contributing to *SA* nasal colonization was elegantly demonstrated in a recent study by Iwase and colleagues in which *Staphylococcus epidermidis*, a resident bacterium of the human nares, was shown to inhibit both nasal colonization and biofilm formation of *SA*. Specifically, they demonstrated that a serine protease (Esp) secreting *S. epidermidis* eliminated *SA* colonizing the nasal cavities of healthy individuals [64]. Perhaps, the absence of Esp-expressing *S. epidermidis* in the nasal niche could potentially contribute to persistent *SA* carriage. Additionally, competitive bacterial interference between *SA* and *Streptococcus pneumoniae* have also been studied extensively. Several studies have confirmed an inverse relationship between *SA* and *S. pneumoniae* colonization in the nasopharyngeal niche [62,65]. This inverse relationship between *SA* and *S. pneumoniae* could influence *SA* carriage.

While we have achieved our goal of assessing the genotypic diversity between *SA* strains from persistent and intermittent carriers, we find it pertinent to note that some inherent limitations complicate data interpretation. This study focused only on nasal carriage strains*,* although *SA* is known to colonize other extra-nasal regions in humans [9]. Regarding the labeling of persistent and intermittent carriers it is important to note that the success rate for isolating *SA* from swab samples never reaches 100%. Moreover, the sample collection was dependent largely on the willingness of donors participating in the study, which lead to gaps in periodicity of sample collection.

## Conclusion

The current study illustrates the lack of genotypic differences in *SA* colonizing persistent and intermittent carriers, and the strain relatedness between these carriers observed within the study may be higher than previously thought. Assessment of nasal carriage dynamics between strains colonizing persistent and intermittent carriers and understanding complex host-pathogen interactions during carriage are crucial for developing effective intervention strategies for nasal carriage and subsequent prevention of community-associated and nosocomial *SA* infections.

## Abbreviations

SA: *Staphylococcus aureus*; MRSA: Methicillin resistant *Staphylococcus aureus*; CA-MRSA: Community-acquired MRSA; MLST: Multi locus sequence typing; SPA: Staphylococcal protein A; ClfB: Clumping factor B

## Competing interests

The authors declare that they have no competing interests.

## Authors’ contribution

GM participated in study conception and design, performed the experiments, genetic analyses, data interpretation, and manuscript preparation. RPL contributed to study design, performed the experiments, provided analytical software, and participated in manuscript preparation. AE VP ABP performed the experiments. ST provided analytical software and helped with data acquisition. CLP participated in data analysis, and manuscript preparation. AMC participated in study conception and design, data interpretation and participated in manuscript preparation. All authors read and approved the final manuscript.

## Pre-publication history

The pre-publication history for this paper can be accessed here:

http://www.biomedcentral.com/1471-2334/13/221/prepub

## Supplementary Material

Additional file 1: Table S1 Complete genotyping details of *S. aureus* strains analyzed in this study. **Table S2***SA* nasal carriage pattern among closely related donors. **Table S3** Nucleotide sequences of SD repeats generated for the gene *clfB*. Click here for file

Additional file 2: Figure S3 Color-coded repeat regions of R domains at the locus *clfB* of all *SA* strains isolated from persistent and intermittent carriers analyzed in this study. Shown here is the nucleotide analysis of the c*lfB* R region on all *SA* strains isolated from persistent (colored in **blue**) and intermittent carriers (colored in **black**). Click here for file

Additional file 3: Figure S1 *SA* strains isolated from nasal carriers are genetically related to nosocomial epidemic strains. Bayesian analyses of *SA* strains isolated from all nasal carriers enrolled in both cross-sectional (with only single nasal culture) and longitudinal studies (persistent carrier strains (**blue**), intermittent carrier strains (**green**)) are genetically similar to *SA* strains isolated from clinical settings (**red**). Numbers at each node indicate posterior probability support and grey-filled circles represent 100% posterior probability. Click here for file

Additional file 4: Figure S2 Longitudinal monitoring of healthy individuals for *SA* nasal carriage also identified true non-carriers of *SA*. Shown here is a representative set of true non-carriers of *SA* that have been monitored for a year or more. (N) indicates *SA* non-carrier state. Click here for file
