# Supplementary material for: Longitudinal genetic analyses of Staphylococcus aureus nasal carriage dynamics in a diverse population
Source: BMC Infect Dis. 2013 May 16;13:221. doi: 10.1186/1471-2334-13-221 (PMC3673815; doi:10.1186/1471-2334-13-221)
Supplement: Additional file 1: Table S1 — Complete genotyping details of S. aureus strains analyzed in this study. Table S2SA nasal carriage pattern among closely related donors. Table S3 Nucleotide sequences of SD repeats generated for the gene clfB. [file 1471-2334-13-221-S1.pdf]

Supplemental Table S1. Complete genotyping details of *S. aureus* strains analyzed in this study

Taxa label	MLST Sequence type (ST)	*spa* type	*spa* clonal complex as revealed by eBURP clustering analysis (*spa*-CC)[a]	*clfB* R region sequence obtained?[a]
D20	59	t216	#7 [b]	YES
D20-2	59	t216	#7	YES
D20-24	5	t688	5: no founder	YES
D20-25	5	t688	5: no founder	YES
D20-3	59	t216	#7	YES
D20-5	1723	t148	4: no founder	YES
D20-6	1723	t148	4: no founder	YES
D20-7	1723	t148	4: no founder	YES
D502-2	106	t056	#1	YES
D502-3	106	t056	#1	YES
D502-4	106	t056	#1	YES
D502-5	106	t056	#1	YES
D502-6	106	t056	#1	YES
D502-7	106	t056	#1	YES
D502-8	106	t056	#1	YES
D502-9	106	t056	#1	YES
D507	582	t084	6: no founder	YES
D507-2	582	t084	6: no founder	YES
D507-3	582	t084	6: no founder	YES
D507-4	582	t084	6: no founder	YES
D512	30	t012	2: *spa*-CC 037	YES
D512-2	30	t012	2: *spa*-CC 037	YES
D512-3	30	t1705	#10	YES
D512-4	30	t012	2: *spa*-CC 037	YES
D512-5	30	t012	2: *spa*-CC 037	YES
D512-7	30	t012	2: *spa*-CC 037	YES
D512-8	30	t012	2: *spa*-CC 037	YES
D512-9	30	t012	2: *spa*-CC 037	YES
D517	8	t012	2: *spa*-CC 037	YES
D517-2	8	t1705	#10	YES
D521	30	t122	2: *spa*-CC 037	YES
D521-2	30	t122	2: *spa*-CC 037	YES
D521-3	8	t036	1: *spa*-CC 024	YES
D521-4	8	t036	1: *spa*-CC 024	YES
D521-5	8	t036	1: *spa*-CC 024	YES
D521-6	8	t036	1: *spa*-CC 024	YES
D521-7	8	t036	1: *spa*-CC 024	YES
D523-10	188	t189	3: no founder	YES
D523-11	188	t189	3: no founder	YES
D523-14	188	t012	2: *spa*-CC 037	YES
D523-5	188	t189	3: no founder	YES
D524[c]	30	NA	NA	NA
D528-10	8	t008	1: *spa*-CC 024	YES
D528-11	8	t008	1: *spa*-CC 024	YES
D528-2	8	t024	1: *spa*-CC 024	YES
D528-3	8	t008	1: *spa*-CC 024	YES
D528-5	8	t008	1: *spa*-CC 024	YES
D528-6	8	t008	1: *spa*-CC 024	YES
D528-7	8	t008	1: *spa*-CC 024	YES

Taxa label	MLST Sequence type (ST)	*spa* type	*spa* clonal complex as revealed by eBURP clustering analysis (*spa*-CC)[a]	*clfB* R region sequence obtained?[a]
D528-8	8	t008	1: *spa*-CC 024	YES
D528-9	8	t008	1: *spa*-CC 024	YES
D531	30	NA	NA	NA
D535	59	t216	#7	YES
D535-10	5	t002	7: no founder	YES
D535-11	5	t002	7: no founder	YES
D535-12	5	t002	7: no founder	YES
D535-13	5	t002	7: no founder	YES
D535-14	5	t002	7: no founder	YES
D535-15	5	t002	7: no founder	YES
D535-16	5	t002	7: no founder	YES
D535-2	30	t216	#7	YES
D535-3	5	t002	7: no founder	YES
D535-4	5	t002	7: no founder	YES
D535-5	5	t002	7: no founder	YES
D535-6	5	t002	7: no founder	YES
D535-7	5	t002	7: no founder	YES
D535-8	5	t002	7: no founder	YES
D535-9	5	t002	7: no founder	YES
D540	15	t346	6:no founder	YES
D540-2	5	NA	NA	YES
D543	5	NA	NA	NA
D547	30	t3263	2: *spa*-CC 037	YES
D547-14	5	t688	5: no founder	YES
D547-15	5	t688	5: no founder	YES
D547-2	1434	t148	4: no founder	YES
D547-3	1507	t002	7: no founder	YES
D547-4	59	t216	#7	YES
D547-5	59	t216	#7	YES
D549-2	8	t008	1: *spa*-CC 024	YES
D549-3	8	t008	1: *spa*-CC 024	YES
D549-4	8	t008	1: *spa*-CC 024	YES
D549-5	8	t008	1: *spa*-CC 024	YES
D553	50	t185	#5	YES
D553-2	50	t185	#5	YES
D553-3	50	t185	#5	YES
D553-4	2224	t185	#5	YES
D554	8	NA	NA	NA
D558	45	NA	NA	NA
D560	508	NA	NA	NA
D563	30	NA	NA	NA
D564	2225	t216	#7	YES
D564-2	2225	t216	#7	YES
D565	87	t216	#7	YES
D565-2	87	t216	#7	YES
D565-3	87	t216	#7	YES
D566	15	t7134	6: no founder	YES
D566-10	30	t037	2: *spa*-CC 037	YES
D566-2	15	t7134	6: no founder	YES
D566-3	30	t037	2: *spa*-CC 037	YES
D566-4	30	t037	2: *spa*-CC 037	YES
D566-5	30	t037	2: *spa*-CC 037	YES

Taxa label	MLST Sequence type (ST)	*spa* type	*spa* clonal complex as revealed by eBURP clustering analysis *(spa*-CC)[a]	*clfB* R region sequence obtained?[a]
D566-6	30	t9877	1: *spa*-CC 024	YES
D566-7	30	t037	2: *spa*-CC 037	YES
D566-8	30	t037	2: *spa*-CC 037	YES
D566-9	30	t037	2: *spa*-CC 037	YES
D574	34	NA	NA	NA
D576	30	t363	2: *spa*-CC 037	YES
D576-2	30	t363	2: *spa*-CC 037	YES
D576-3	30	t363	2: *spa*-CC 037	YES
D576-4	30	t363	2: *spa*-CC 037	YES
D577	672	NA	NA	NA
D579	398	NA	NA	NA
D582	5	t002	7: no founder	YES
D582-2	1656	t216	#7	YES
D582-3	5	t002	7: no founder	YES
D584	45	NA	NA	NA
D589	45	t9652	#12	YES
D589-2	45	t9652	#12	YES
D589-3	45	t9652	#12	YES
D592	30	NA	NA	NA
D594	188	t189	3: no founder	YES
D594-2	188	t189	3: no founder	YES
D594-3	188	t189	3: no founder	YES
D594-4	188	t189	3: no founder	YES
D594-5	188	t037	2: *spa*-CC 037	YES
D594-6	188	t189	3: no founder	YES
D597	15	NA	NA	NA
D599	30	t037	2: *spa*-CC 037	YES
D599-9	398	t037	2: *spa*-CC 037	YES
D604-5	5	t688	5: no founder	YES
D604-6	5	t688	5: no founder	YES
D605	1181	t334	1: *spa*-CC 024	YES
D607	30	NA	NA	NA
D608	30	NA	NA	NA
D613	97	t1247	#9	YES
D618	5	t954	5: no founder	YES
D618-3	2226	t954	5: no founder	YES
D618-4	5	t954	5: no founder	YES
D619	5	t954	5: no founder	YES
D619-2	5	t954	5: no founder	YES
D619-3	5	t954	5: no founder	YES
D619-5	5	t954	5: no founder	YES
D619-6	5	t954	5: no founder	YES
D619-7	5	t954	5: no founder	YES
D623	5	t688	5: no founder	YES
D623-2	5	t688	5: no founder	YES
D623-3	5	t688	5: no founder	YES
D623-4	5	t688	5: no founder	YES
D627	15	t1509	Excluded	YES
D627-2	1659	t1509	Excluded	YES
D628	109	NA	NA	NA
D629	109	t3745	Excluded	YES
D629-2	109	t3745	Excluded	YES

Taxa label	MLST Sequence type (ST)	*spa* type	*spa* clonal complex as revealed by eBURP clustering analysis *(spa*-CC)[a]	*clfB* R region sequence obtained?[a]
D635	5	t954	5: no founder	YES
D635-2	5	t954	5: no founder	YES
D635-3	5	t954	5: no founder	YES
D636	1658	t021	2: *spa*-CC 037	YES
D636-2	1658	t021	2: *spa*-CC 037	YES
D637	8	t2648	1: *spa*-CC 024	YES
D637-2	8	t2648	1: *spa*-CC 024	YES
D637-4	8	t2648	1: *spa*-CC 024	YES
D637-7	8	t2648	1: *spa*-CC 024	YES
D643	508	NA	NA	NA
D647-2	87	t216	#7	YES
D647-5	87	t216	#7	YES
D647-7	87	t216	#7	YES
D647-8	87	t216	#7	YES
D651	30	t338	2: *spa*-CC 037	YES
D655-3	30	t338	2: *spa*-CC 037	YES
D657	45	t9876	#13	YES
D657-2	2228	t9876	#13	YES
D662	30	t021	2: *spa*-CC 037	YES
D662-2	30	t021	2: *spa*-CC 037	YES
D664	72	t3682	4: no founder	YES
D664-2	72	t3682	4: no founder	YES
D664-3	72	t3682	4: no founder	YES
D672	188	t9873	3: no founder	YES
D672-10	188	t9873	3: no founder	YES
D672-12	188	t9873	3: no founder	YES
D672-16	188	t9873	3: no founder	YES
D672-2	1724	t9873	3: no founder	YES
D672-3	188	t9873	3: no founder	YES
D672-4	188	t9873	3: no founder	YES
D672-5	188	t9873	3: no founder	YES
D672-6	188	t9873	3: no founder	YES
D672-7	188	t9873	3: no founder	YES
D672-8	188	t9873	3: no founder	YES
D672-9	188	t9873	3: no founder	YES
D678	109	NA	NA	NA
D681	1159	t091	#3	YES
D681-10	1159	t091	#3	YES
D681-11	1159	t091	#3	YES
D681-12	1159	t091	#3	YES
D681-2	1159	t091	#3	YES
D681-3	1159	t091	#3	YES
D681-4	1159	t091	#3	YES
D681-5	1159	t091	#3	YES
D681-6	1159	t091	#3	YES
D681-7	1159	t091	#3	YES
D681-8	1159	t091	#3	YES
D681-9	1159	t091	#3	YES
D686-2	2229	t701	1: *spa*-CC 024	YES
D691	30	NA	NA	NA
D692	30	t012	2: *spa*-CC 037	YES
D692-2	2230	t209	#6	YES

Taxa label	MLST Sequence type (ST)	*spa* type	*spa* clonal complex as revealed by eBURP clustering analysis (*spa*-CC)[a]	*clfB* R region sequence obtained?[a]
D692-3	8	t2229	#11	YES
D692-4	30	t012	2: *spa*-CC 037	YES
D697	109	t209	#6	YES
D697-2	109	t209	#6	YES
D710	30	NA	NA	NA
D713-4	5	t548	7: no founder	YES
D714	81	t127	#4	YES
D714-4	81	t127	#4	YES
D714-5	81	t127	#4	YES
D714-6	81	t127	#4	YES
D719	30	NA	NA	NA
D720	1657	t1001	#8	YES
D720-2	1657	t1001	#8	YES
D720-3	1657	t1001	#8	YES
D720-4	1657	t1001	#8	YES
D720-5	1657	t1001	#8	YES
D720-6	1657	t1001	#8	YES
D720-7	1657	t1001	#8	YES
D720-8	1657	t1001	#8	YES
D720-9	1657	t1001	#8	YES
D724	45	t073	#2	YES
D724-2	2231	t073	#2	YES
D724-3	45	t073	#2	YES
D724-4	45	t073	#2	YES
D724-5	45	t073	#2	YES
D724-6	45	t073	#2	YES
D724-7	45	t073	#2	YES
D724-8	45	t073	#2	YES
D725-2	5	t002	7: no founder	YES
D725-3	5	t002	7: no founder	YES
D729	5	NA	NA	NA
D732	5	NA	NA	NA
D733	5	NA	NA	NA
D735-2	8	t3240	1: *spa*-CC 024	YES
D739-2	30	t8072	2: *spa*-CC 037	YES
D742	1181	t334	1: *spa*-CC 024	YES
D742-2	1181	t334	1: *spa*-CC 024	YES
D750	72	t3682	4: no founder	YES
D750-2	72	t3682	4: no founder	YES
D752	8	NA	NA	NA
D753-5	2232	NA	NA	YES
D756	2227	t012	2: *spa*-CC 037	YES
D756-2	2227	t012	2: *spa*-CC 037	YES
D756-3	2227	t012	2: *spa*-CC 037	YES
D757-5	8	t008	1: *spa*-CC 024	YES
D758	15	NA	NA	NA
D771	8	t008	1: *spa*-CC 024	YES
D771-2	8	t008	1: *spa*-CC 024	YES
D771-3	8	t008	1: *spa*-CC 024	YES
D776	508	NA	NA	NA
D785-2	8	NA	NA	NA
D785-4	8	t008	1: *spa*-CC 024	YES

Taxa label	MLST Sequence type (ST)	*spa* type	*spa* clonal complex as revealed by eBURP clustering analysis *(spa*-CC)[a]	*clfB* R region sequence obtained?[a]
D795	15	t084	6: no founder	YES
D795-2	15	t084	6: no founder	YES
D795-4	15	t084	6: no founder	YES
D795-5	15	t084	6: no founder	YES
D795-6	15	t084	6: no founder	YES
D795-7	15	t084	6: no founder	YES
D798	8	t008	1: *spa*-CC 024	YES
D798-2	8	t008	1: *spa*-CC 024	YES
D798-3	8	t008	1: *spa*-CC 024	YES
D798-4	8	t008	1: *spa*-CC 024	YES
D798-5	8	t008	1: *spa*-CC 024	YES
D798-6	8	t008	1: *spa*-CC 024	YES
D798-7	8	t008	1: *spa*-CC 024	YES
D799	5	NA	NA	NA
D812-3	716	t008	1: *spa*-CC 024	YES
D819	30	NA	NA	NA
D506-5[#]	NA	NA	NA	NA

[#] No ST available.
[a] *spa* typing and *clfB* typing was performed on *S. aureus* strains isolated from carriers enrolled in longitudinal analysis study (i.e. nasal swabs from carriers monitored 2 or more times).
[b] Cluster number to which the strain belongs as revealed by *spa* eBURP clustering analysis. Founder *spa* type, if present, for each *spa*-CC is also shown here.
[c] Italicized taxa = Cross sectional donors monitored only once to detect *S. aureus* in the nares.
NA = sequence type or *spa* type or clfB typing information not available.

Supplemental Table S2. *SA* nasal carriage pattern among closely related donors

Closely related donors	Type of donor relationship	Total *SA* strains	Identical *SA* strains as classified by MLST	Non-identical SA strains as classified by MLST
**D528-D549**	Spouse	13	13	0
**D523-D594**	Spouse	10	10	0
**D618-D619**	Living together	10	9	1
**D619-D635**	Twins	10	10	0
**D20-D547-D604**	Father-Mother-Child	18	12	6

Supplemental Table S3. Nucleotide sequences of SD repeats generated for the gene *clfB*

Repeat numbers	Repeat sequences	Repeat numbers	Repeat sequences
1	TCGGATTCGGACAGTGAC	50	TCGGATTCAAACAGCGAT
2	TCAGGCTCAGACAGCGAC	51	TCGGACTCAGACAGTGAC
3	TCAGGTTCAGACAGTGAC	52	TCAAACTCAGATAGTGAC
4	TCGGACTCAGACAGCGAC	53	TCGGATTCAGATAGCGAT
5	TCAGATTCAGATAGTGAC	54	TCGGATTCAGATAGCGAC
6	TCAGACTCAGATAGTGAC	55	TCAGACCCAGACAGTGAG
7	TCAGATTCAGACAGCGAT	56	TCAGATTCAGACAGTGAG
8	TCGGATTTAGACAGCGAT	57	TCAGACTCGGATAGCGAT
9	TCGGATTCAGACAGCGAC	58	TCGGACTCAGACAGTTAC
10	TCAGATTCAGATAGTGAT	59	TCAGGTTCAGACAGTGAG
11	TCAGATTCAGACAGCGAC	60	TCGGGTTCAGATAGCGAC
12	TCAGACTCAGATAGTGAT	61	TCGGAATCAGACAGTGAT
13	TCAGACTCAGACAGTGAG	62	TCAGATTCCGACAGCGAC
14	TCAGATTCAGATAGCGAT	63	TCGGACTCAGATAGCAAC
15	TCAGACTCAGACAGTGAC	64	TCGGATTCGGACAGCGAC
16	TCCGATTCAGATAGCGAT	65	ACAGATTCAGATAGTGAC
17	TCGGACTCAGATAGCGAC	66	ACAGATTCAGACAGCGAC
18	TCCGATTCAGATAGCGAG	67	TCTGATTCAGACAGCGAC
19	TCAGACTCAGACAGTGAT	68	TCCGATTCAGATAGTGAT
20	TCGGATTCAGACAGCGAT	69	TCCGACACGGACAGCGAC
21	TCGGATTCAGACAGTGAC	70	TCAGATTCAGAAAGTGAC
22	TCAGGTTCAGATAGCGAC	71	TCCGATTCAGACAGCGAT
23	TCAGAATCAGATAGCGAT	72	TCTGATTCAGACAGCGAT
24	TCGGATTCAGACAGTGAT	73	TCAGATTCAGAGAGCGAT
25	TCAGAATCAGATAGCGAC	74	TCCGACTCAGACAGCGAC
26	TCAGAATCAGATAGTGAG	75	TCCGGTTCAGATAGTGAT
27	TCAGATTCAGACAGTGAC	76	TCAGATTCCGACAGCGAT
28	TCGGACTCAGACAGTGAT	77	TCGGATTCCGACAGCGAC
29	TCAGACTCAGATAGCGAT	78	TCAGATTCCGACAGTGAT
30	TCAGACTCAGACAGCGAT	79	TCCGACTCAGACAGCGAT
31	TCAGAATCAGACAGCGAC	80	TCCGATTCAGATAATGAC
32	TCAGACTCAGATAGCGAC	81	TCCGATTCTGATAGTGAC
33	TCAGACTCAGACAGCGAC	82	TCCGACTCTGATAGTGAC

Repeat numbers	Repeat sequences	Repeat numbers	Repeat sequences
34	TCGGACTCAGACAGCGAT	83	TCTGATTCAGATAGTGAT
35	TCAGACTCGGATAGCGAC	84	TCCGATTCAGACAGTGAC
36	TCGGATTCAGATAGTGAC	85	TCAGACTCAGAAAGCGAT
37	TCAGAATCAGACAGTGAT	86	TCACACTCAGATAGTGAC
38	TCAGGTTCAGATAGCGAT	87	TCGGACTCGGATAGTGAC
39	TCAGATTCAGATAGCGAC	88	TCAGACTCAGGTAGCGAT
40	TCAGAATCAGACAGTGAC	89	TCAGACTCAGATAGTGAG
41	TCAGACAGCGAT	90	TCAGATTCAGACAGCGAG
42	TCAGATTCAGATAGTGAG	91	TCGGATTCCGACAGTGAT
43	TCGGACTCAGATAGCGAT	92	TCAGATTCAGATAGCAAT
44	TCGGATTCAGACAACGAT	93	TCAGAGTCAGATAGTGAG
45	TCAGAATCAGACAGCGAT	94	TCAGATTCGGACAGCGAT
46	TCAGAATCAGACAGTGAG	95	TCAGATTCAGATAGCAAC
47	TCAAACTCAGACAGTGAG	96	TCGAATTCAGACAGTGAT
48	TCGGACTCAGATAGTGAC	97	TCAGACTCATACAGTGAT
49	TCGGACTCAGACAGTGAG	98	TCAGATTCAGGTAGTGAC
99	TCAGATTCCGATAGTGAC	114	TCGGACTCAGAGAGCGAT
100	TCCGACTCCGACAGCGAT	115	TCAGATTCAGACGGCGAT
101	TTAGATTCAGATAGCGAT	116	TCGGAGTCAGATAGCGAC
102	TCAGGCTCAGACAGCGAT	117	TCGGACTCAGACAGTGAA
103	TCGGATTCAGACAGTGAG	118	TCAGAATCAGACGGCGAT
104	TCAGGTTCCGATAGCGAT	119	TCAGACTCGTGTAGCGAT
105	TCGGATTCCGATAGTGAC	120	TCCGACTCAGGTAGCTGT
106	TCCGACTCAGATAGTGAC	121	TCAGACTCCGATAGTGAG
107	TCGGAGTCAGAGAGTGAC	122	TCAGATTCTTACAGCGAT
108	TCAGACTCTTATAGTGAC	123	TCCCACTCAGGTAGCAAT
109	TCGGACTCAGAAAGTGAC	124	TCAGACAGTGAC
110	TCGGACTCGGACTGTGAA	125	TCAGATTCAGACGGCGAC
111	TCGGATTCAAAGAGCGAT	126	TCCGATTCAGATAGCGAC
112	TCGGATTCAGACAGAGAC	127	TCAGACTCCGACAGCGAT
113	TCGGACGCAGATACCGAC		
