# Supplementary figures and images for: Longitudinal genetic analyses of Staphylococcus aureus nasal carriage dynamics in a diverse population
Source: BMC Infect Dis. 2013 May 16;13:221. doi: 10.1186/1471-2334-13-221 (PMC3673815; doi:10.1186/1471-2334-13-221)

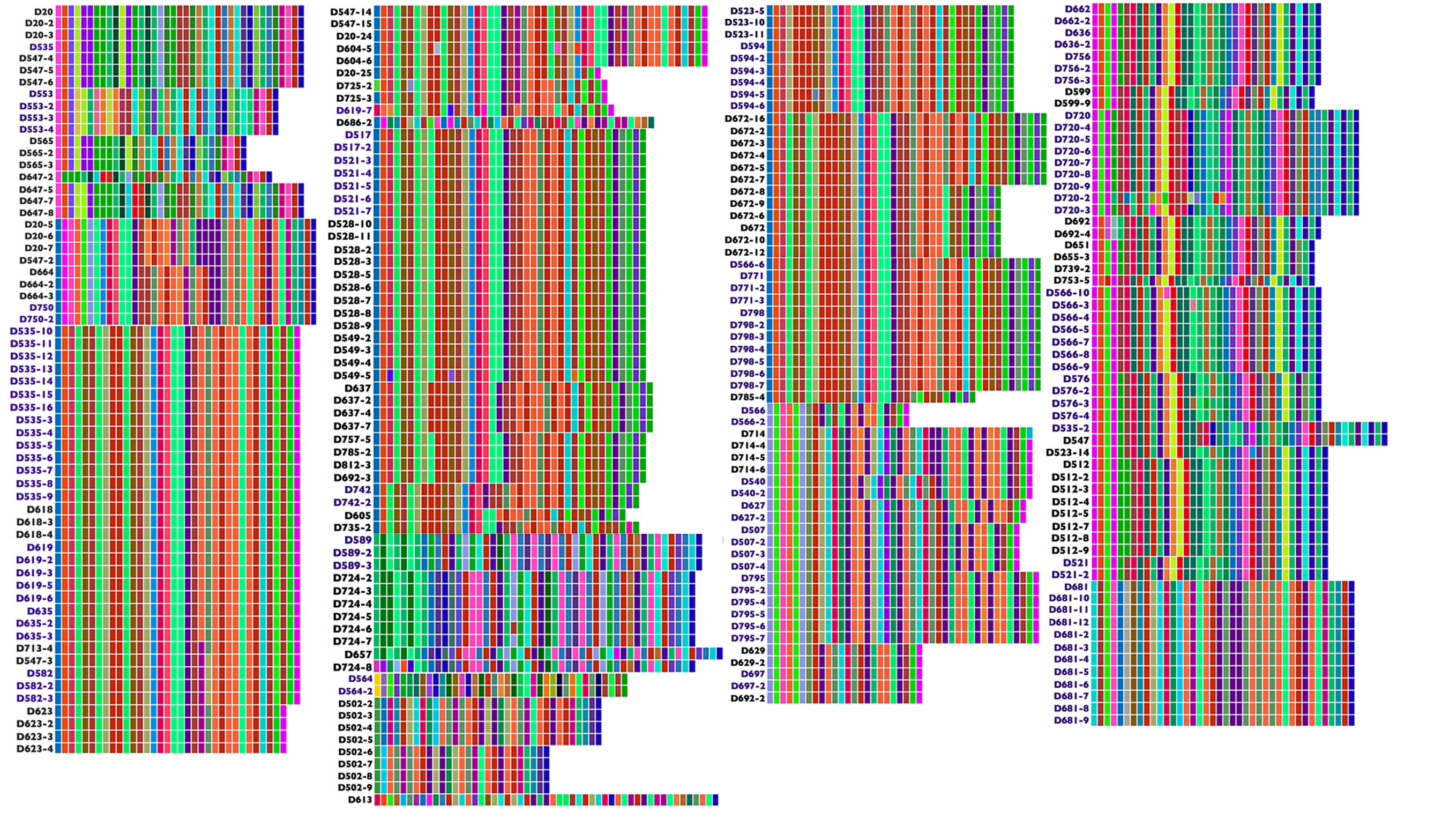

Supplement: Additional file 2: Figure S3 — Color-coded repeat regions of R domains at the locus clfB of all SA strains isolated from persistent and intermittent carriers analyzed in this study. Shown here is the nucleotide analysis of the clfB R region on all SA strains isolated from persistent (colored in blue) and intermittent carriers (colored in black). [file 1471-2334-13-221-S2.tiff]

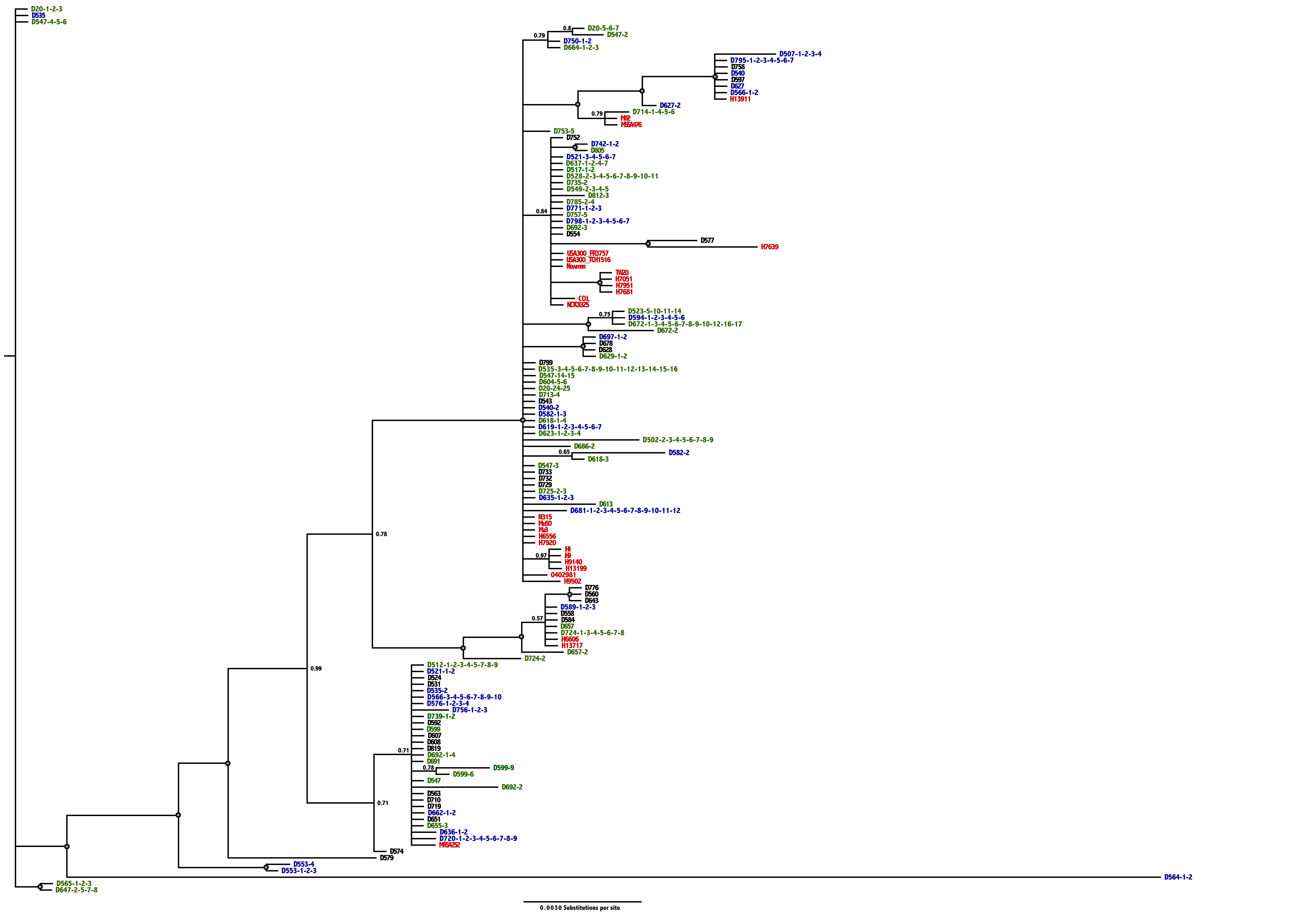

Supplement: Additional file 3: Figure S1 — SA strains isolated from nasal carriers are genetically related to nosocomial epidemic strains. Bayesian analyses of SA strains isolated from all nasal carriers enrolled in both cross-sectional (with only single nasal culture) and longitudinal studies (persistent carrier strains (blue), intermittent carrier strains (green)) are genetically similar to SA strains isolated from clinical settings (red). Numbers at each node indicate posterior probability support and grey-filled circles represent 100% posterior probability. [file 1471-2334-13-221-S3.tiff]

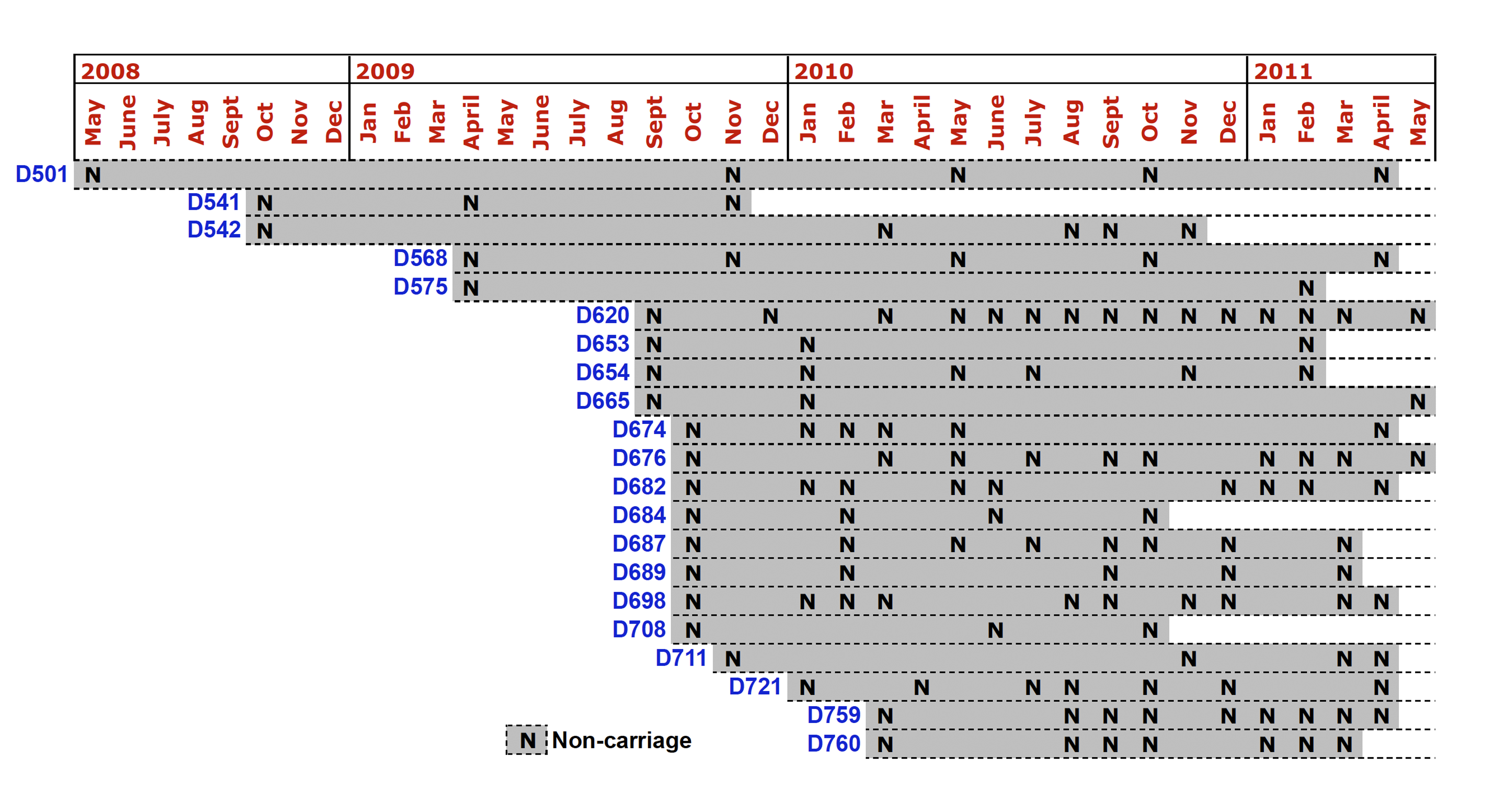

Supplement: Additional file 4: Figure S2 — Longitudinal monitoring of healthy individuals for SA nasal carriage also identified true non-carriers of SA. Shown here is a representative set of true non-carriers of SA that have been monitored for a year or more. (N) indicates SA non-carrier state. [file 1471-2334-13-221-S4.tiff]
